# Disruption of temporo-parietal network in Alzheimer’s disease and its association with memory impairment

**DOI:** 10.1186/s13195-026-02138-w

**Published:** 2026-07-15

**Authors:** Yanin Suksangkharn, Björn Hendrik Schott, Peter Zeidman, Niklas Vockert, René Lattmann, Hartmut Schütze, Renat Yakupov, Oliver Peters, Julian Hellmann-Regen, Lukas Preis, Ersin Ersözlü, Josef Priller, Eike Jakob Spruth, Janna Beckmann, Anja Schneider, Klaus Fliessbach, Jens Wiltfang, Claudia Bartels, Ayda Rostamzadeh, Wenzel Glanz, Enise I. Incesoy, Stefan Teipel, Ingo Kilimann, Doreen Goerss, Christoph Laske, Annika Spottke, Marie Kronmüller, Frederic Brosseron, Falk Lüsebrink, Matthias Schmid, Luca Kleineidam, Melina Stark, Stefan Hetzer, Peter Dechent, Frank Jessen, Anne Maass, Emrah Düzel, Gabriel Ziegler

**Affiliations:** 1 German Center for Neurodegenerative Diseases (DZNE), Magdeburg, 39120 Germany; 2 Institute of Cognitive Neurology and Dementia Research (IKND), Otto-von- Guericke University, Leipziger Str. 44, Magdeburg, 39120 Germany; 3 German Center for Neurodegenerative Diseases (DZNE), Göttingen, 37075 Germany; 4 Department of Psychiatry and Psychotherapy, University Medical Center Goettingen, University of Goettingen, Von-Siebold-Str. 5, Goettingen, 37075 Germany; 5 Department of Psychiatry and Psychotherapy, University Hospital Magdeburg, Otto-von-Guericke University, Magdeburg, 39120 Germany; 6 Center for Behavioral Brain Sciences (CBBS), Magdeburg, 39106 Germany; 7 Functional Imaging Laboratory, UCL Department of Imaging Neuroscience, 12 Queen Square, London, WC1N 3AR UK; 8 German Center for Neurodegenerative Diseases (DZNE), Berlin, 10117 Germany; 9 Department of Psychiatry and Neurosciences, Charité Universitätsmedizin Berlin, Hindenburgdamm 30, Berlin, 12203 Germany; 10 ECRC Experimental and Clinical Research Center, Charité Universitätsmedizin Berlin, Lindenberger Weg 80, Berlin, 13125 Germany; 11 Neuropsychiatry, Department of Psychiatry and Psychotherapy, Charité, Charitéplatz 1, Berlin, 10117 Germany; 12 University of Edinburgh and UK DRI, Edinburgh, UK; 13 Department of Psychiatry and Psychotherapy, School of Medicine and Health, Technical University of Munich, German Center for Mental Health (DZPG), Munich, Germany; 14 German Center for Neurodegenerative Diseases (DZNE), Bonn/Cologne, Venusberg-Campus 1, Bonn, 53127 Germany; 15 Department of Old Age Psychiatry and Cognitive Disorders, University Hospital Bonn, University of Bonn, Bonn, Germany; 16 Department of Medical Sciences, Neurosciences and Signaling Group, Institute of Biomedicine (iBiMED), University of Aveiro, Aveiro, Portugal; 17 Department of Psychiatry, Medical Faculty, University of Cologne, Kerpener Strasse 62, Cologne, 50924 Germany; 18 Department of Psychiatry, Psychotherapy, and Psychosomatics, Vivantes Klinikum Am Urban, Vivantes Urban Hospital, Berlin, Germany; 19 German Center for Neurodegenerative Diseases (DZNE), Rostock, Germany; 20 Department of Psychosomatic Medicine, Rostock University Medical Center, Gehlsheimer Str. 20, Rostock, 18147 Germany; 21 German Center for Neurodegenerative Diseases (DZNE), Tübingen, Germany; 22 Section for Dementia Research, Hertie Institute for Clinical Brain Research, Department of Psychiatry and Psychotherapy, University of Tübingen, Tübingen, Germany; 23 Department of Neurology, University of Bonn, Venusberg-Campus 1, Bonn, 53127 Germany; 24 Institute for Medical Biometry, Informatics and Epidemiology, University Hospital Bonn, Venusberg-Campus 1, Bonn, D-53127 Germany; 25 Department for Cognitive Disorders and Old Age Psychiatry, University Hospital Bonn, Bonn, Germany; 26 Berlin Center for Advanced Neuroimaging, Charité – Universitätsmedizin Berlin, Berlin, Germany; 27 MR-Research in Neurosciences, Department of Cognitive Neurology, University Medical Center Goettingen, Georg-August-University Goettingen, Goettingen, Germany; 28 Excellence Cluster on Cellular Stress Responses in Aging-Associated Diseases (CECAD), University of Cologne, Joseph-Stelzmann-Strasse 26, Köln, 50931 Germany; 29 Faculty of Natural Sciences, Otto von Guericke University Magdeburg, Magdeburg, Germany

**Keywords:** Alzheimer's disease, Memory impairment, Synaptic aberration, Beta-amyloid, Tau, Neurodegeneration

## Abstract

Alzheimer’s disease (AD) is characterised by the accumulation of β-amyloid (Aβ) and tau proteins, resulting in neurodegeneration and cognitive decline. Although Aβ and tau disrupt synaptic function, the association linking these molecular pathologies to network-level dysfunction and memory impairment remains poorly understood. Here, we investigated the effects of Aβ and tau pathology (CSF Aβ42/40 ratio and tau phosphorylated at position 181, p-tau-181, respectively) on effective connectivity related to memory encoding, which may provide a link between synaptic pathology and cognitive outcomes. Functional magnetic resonance imaging (fMRI) during visual memory encoding was acquired from 205 participants in the multicentric DZNE Longitudinal Cognitive Impairment and Dementia Study (DELCODE) across the AD spectrum. Effective connectivity was assessed using Dynamic Causal Modelling (DCM) of task-fMRI data, focusing on the parahippocampal place area (PPA), hippocampus (HC), and precuneus (PCU)—regions central to memory encoding. Disruptions in connectivity between temporal and parietal lobes were associated with both memory impairment and indices of AD pathology. Specifically, reduced positive effective connectivity from the PCU to the PPA and from the HC to the PCU were linked to higher p-tau-181 levels, with an amplification effect observed in the presence of amyloid accumulation for the latter connectivity. The disruption from the PCU to the PPA was found to be associated with decreased memory performance. Together, these findings indicate that temporo-parietal connectivity is associated with both AD molecular pathology and, for a subset of connections, with memory performance.

**Supplementary Information:**

The online version contains supplementary material available at 10.1186/s13195-026-02138-w.

## Introduction

As Alzheimer’s disease (AD) progresses in the brain, it causes cognitive impairment and decline due to neurodegeneration and synaptic dysfunction. Impairment and functional decline in AD do not only depend on which brain regions are affected but also on the integrity and dynamics of interactions among these regions. However there is still a lack of studies investigating how connectivity across brain regions of the episodic memory network relates to AD pathology and cognitive impairment during the execution of memory tasks.

AD is characterised by spatially complex aggregation of beta-amyloid (Aβ) plaques and tau protein, both contributing synergistically to synaptic dysfunction, neuronal loss, and cognitive decline [1, 2]. Aβ accumulation begins in neocortical areas, before progressing to deeper brain structures [3, 4]. Whereas tau accumulation follows a different anatomical spreading pattern: it often originates in the brainstem and spreads to limbic regions critical for memory and spatial navigation, most notably the entorhinal cortex and hippocampal formation [5, 6]. Recent evidence also suggests that Aβ-induced hyperconnectivity facilitates the spread of tau pathology across functionally connected brain regions, particularly to Aβ-accumulating regions, further exacerbating synaptic disruption and cognitive decline [7, 8]. The distinct spatial trajectories and directionally asymmetric interactions contribute to region-specific vulnerabilities [9]. Understanding how these pathologies interact with network-level dynamics of human memory function can provide insights into the pathological mechanisms of AD.

Dynamic Causal Modelling (DCM) is a frequently used approach to study network-level dynamics. It possesses several advantages compared to other techniques, notably biological interpretability, such as non-linear interactions and biophysical constraints [10, 11]. In the context of fMRI, DCM incorporates both a neural and a haemodynamic model and employs a generative approach to estimate the effective connectivity of the neural model, which can approximate synaptic connectivity at the population level. Thus, it informs the bidirectional relationships between regions and the excitatory-inhibitory balance within each [12, 13].

While DCM has advanced our understanding of effective connectivity related to memory processes [14, 15] and their age-related alterations [16, 17], its application to ageing and AD remains sparse. In ageing, there is evidence for altered effective connectivity between medial temporal and parietal lobes in older compared with younger adults during visual memory encoding [17]. Moreover, in AD, amyloid and tau pathology have been shown to affect the hyperexcitability between the default mode network and medial temporal lobe in a repetition suppression paradigm [9]. However, there remain unaddressed critical gaps. First, the specific alterations in the memory-encoding network, which is prominently affected by AD pathology [18], have yet to be systematically investigated. Second, the inherently non-linear relationships among AD pathology, functional connectivity, and cognitive decline must be considered [1, 19–21].

**Fig. 1:**
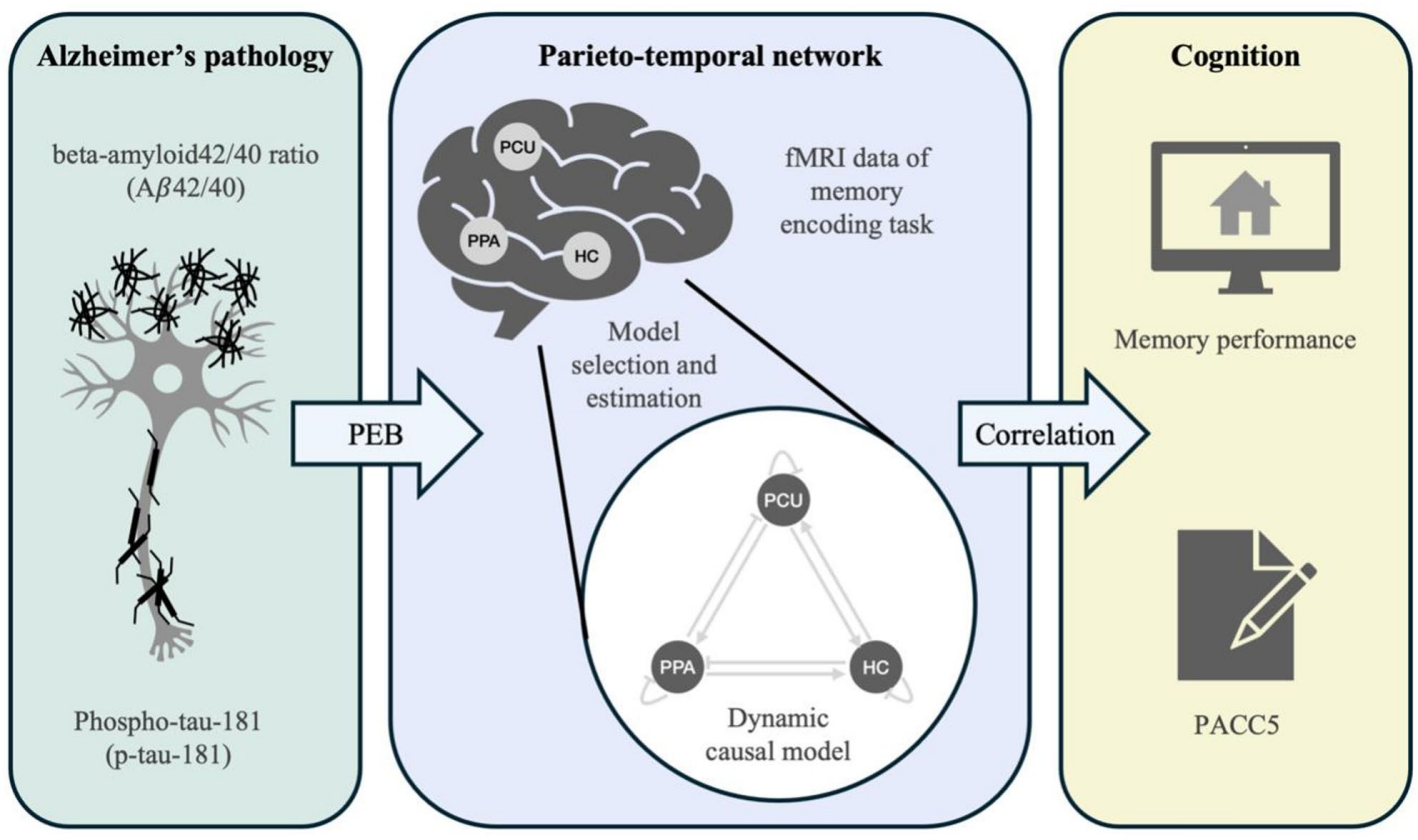
Schematic overview of the study procedures. (Left) Alzheimer’s pathology includes amyloid-β42/40 ratio (Aβ42/40) and phospho-tau181 (p-tau-181) from cerebrospinal fluid (CSF). Parametric empirical Bayes (PEB) was used to investigate the relationship between pathology and fMRI memory-encoding network dynamics. (Middle) Functional network dynamics during a memory-encoding task were modelled using Dynamic Causal Modelling (DCM) of the fMRI time series with regions of interest: parahippocampal place area (PPA), hippocampus (HC), and precuneus (PCU). DCM group-model selection and estimation were performed using SPM12. (Right) Cognition, as the clinical presentation, was studied using memory performance during the task-based fMRI session and the clinical PACC5 composite score. The correlation between connectivity parameters and cognition was evaluated

The present study aimed to evaluate altered effective connectivity within the memory-encoding network as a function of AD pathology and its role in memory impairment. We assessed task-related effective connectivity within the memory-encoding network. We focused on the temporo-parietal network —comprising the hippocampus (HC), the parahippocampal place area (PPA), and the precuneus (PCU) —because these structures are among the earliest and most consistently affected by AD pathology, particularly tau deposition in the medial temporal lobe and amyloid accumulation in parietal cortex [5, 18, 39]. Our approach involved two main analyses (Fig. 1): First, we examined the effects of amyloid and tau on effective connectivity, taking their interaction into account; Second, we explored the link between altered effective connectivity and episodic memory deficits in Alzheimer’s disease. To this end, we analysed task fMRI data from a large cohort of 205 older adults spanning the AD spectrum, including cognitively normal individuals (CN), those with subjective cognitive decline (SCD), mild cognitive impairment (MCI), and early-stage dementia of Alzheimer’s type (DAT), focusing on a visual memory-encoding task previously established in individuals at risk of AD [19, 21, 22]. Based on a previous DCM study using the same paradigm in an independent ageing cohort17, we focus on the HC, the PPA, and the PCU as regions of interest. We hypothesised that effective connectivity in the temporo-parietal network would be associated with amyloid and tau burden, their potential interactions, and that these connectivity disruptions would relate to individual differences in memory impairment.

## Materials and methods

In this study, we examined the relationship between measures of Alzheimer’s pathology, functional network dynamics, and cognitive performance. The following sections describe the rationale, procedures, and methods.

### Study cohort

The study sample comprised participants from the DZNE Longitudinal Cognitive Impairment and Dementia Study (DELCODE), a memory-clinic-based multicenter cohort conducted by the German Centre for Neurodegenerative Diseases (DZNE) (for details, see Jessen et al. [23]). Among 1,079 enrolled participants, 558 individuals completed the fMRI visual memory-encoding task. Following outlier removal and quality-control procedures (see “Participant exclusion”), the final sample comprised 492 participants, of whom 235 had available CSF biomarker data, and 205 were part of ATN classification of interest (see “Characterisation of Alzheimer’s pathology and memory performance”). The study protocol was approved by the local institutional review boards of all participating sites. All participants provided written informed consent in accordance with the Declaration of Helsinki. The DELCODE study was retrospectively registered with the German Clinical Trials Register (DRKS00007966) on 4 May 2015. Details on data handling and quality control have been described previously [23].

### Experimental paradigm

Participants underwent fMRI scanning while performing a visual novelty-encoding task, viewing 44 novel indoor scenes, 44 novel outdoor scenes, and 44 repetitions of pre-familiarised images. They classified each image as *indoor* or *outdoor* scene via button press. Each stimulus was displayed for 2500 ms, followed by fixation with an optimised jitter ranging from 0.70 s to 2.65 s [24]. The task lasted approximately 11 min, during which 206 functional volumes were recorded. After a 90-minute delay, a computer-based recognition-memory test was conducted outside the scanner to assess recognition of the novel images seen in the fMRI session. The test consisted of 88 previously seen novel images and 44 new images, using a 5-step response scale where 1 = “definitely new” and 5 = “definitely old”. The recognition responses were converted into a parametric modulator with an arcsine-transformation

$$\:PM=\mathrm{arcsin}\left(\frac{x-3}{2}\right)\cdot\:\frac{2}{{\uppi\:}}$$

as this approach has previously been shown to outperform categorical models for the same task [25]. The responses were used as predictor during first-level analysis (denoted as the subsequent memory score). Memory performance for each participant was quantified by the area under the curve (AUC) of hits (correctly recognised novelty stimuli) against false alarms (incorrectly recognised novelty stimuli), taking the subsequent-memory score into account (hence termed A’; see Soch et al. [26]). An A’ of 0.5 reflects chance performance, whereas 1 reflects perfect recognition.

### Characterisation of Alzheimer’s pathology and memory performance

AD pathology was characterized according to the NIA-AA research framework [27], encompassing amyloid accumulation (A), pathological tau accumulation (T), and neurodegeneration (N). We employed the CSF Aβ42/40 ratio and phosphorylated tau at position 181 (p-tau-181) as core biomarkers. The Aβ42/40 ratio was selected over Aβ42 alone because it corrects for inter-individual differences in total amyloid production and CSF dynamics, yielding superior concordance with amyloid PET30 and improved diagnostic accuracy in differentiating AD from other dementias [28]. CSF p-tau-181 was chosen as the established tau marker within the DELCODE protocol because of its well-documented sensitivity to early tau-related changes and its widespread use in large-cohort AD research [28, 29]. Binary classifications (A-/A+, T-/T+, and N-/N+) were applied using cut-offs determined from the DELCODE cohort via Gaussian-mixture modelling: Aβ42/40 $\:\le\:$ 0.08 pg/ml for A+, p-tau-181 $\:\ge\:\:$73.65 pg/ml for T+, and adjusted hippocampal volume $\:\le\:\mathrm{2,821.1}\:\mu\:l\:for\:N+\:$(see Düzel et al. [20] and Heinzinger et al. [28]). As AD spectrum is defined as sequential transitions of A, T, and N, we only selected 205 individuals that were categorized as A-T-N-, A + T-N-, and A + T+N- in order to evaluate the effects of amyloid and tau, while discarding A-T-N+ in order to control for N effect.

Cognitive outcomes were assessed using memory performance (A’) from the memory-encoding fMRI task (see previous section). In addition, the Preclinical Alzheimer’s Cognitive Composite 5 (PACC5) [29] was included as an independent validation measure. PACC5 is a composite score derived from the MMSE, the summed Free and Cued Selective Reminding Test (FCSRT) free and total recall, the Wechsler Memory Scale – Fourth Edition (WMS-IV) Logical Memory Story B delayed recall, the Symbol-Digit Modalities Test (SDMT), and the sum of two category-fluency tasks, which together predict early disease progression. This renders PACC5 particularly sensitive to detecting preclinical changes within the AD spectrum [30].

### MRI acquisition and MRI data processing

MRI data were acquired using 3T Siemens scanners across participating sites including TIM Trio, Verio, Skyra, and Prisma systems. To mitigate potential scanner-related batch effects in this multicentre design, acquisition protocols were harmonised across sites. Structural images included T1-weighted (1 $\:{mm}^{3}$ isotropic resolution) and T2-weighted acquisitions optimised for medial temporal lobe volumetry. Functional images were obtained using a T2*-weighted echo-planar imaging (EPI) sequence (TR = 2580 ms, TE = 30 ms, voxel size = 3.5 mm isotropic). Hippocampal volumes were extracted using the FreeSurfer longitudinal processing stream (version 6.0) [31–33], which performs automated subcortical segmentation on the T1-weighted structural images. Total hippocampal volume was computed as the sum of left and right hippocampal grey matter volumes from the FreeSurfer aseg output. Finally, adjusted hippocampal volume was derived from the hippocampal volume corrected for age, sex, education, total intracranial volume (TICV), and WMH using a linear regression model.

Preprocessing of fMRI data was conducted using SPM12 and involved multiple steps: correction for acquisition delay (slice-timing), head motion (realignment), and magnetic-field inhomogeneities (unwarping; using individual phase and magnitude fieldmaps); followed by normalisation to a population standard space with geodesic shooting nonlinear registration [34, 35], normalisation into a standard stereotactic reference space (Montreal Neurological Institute (MNI), voxel size = 3.5 mm isotropic), and spatial smoothing (Gaussian kernel, FWHM = 6 mm).

To construct a DCM of functional dynamics during memory encoding, we specified first-level GLMs including onsets of novel and non-novel scenes and a parametric modulator of recognition responses, as suggested in a previous model-selection study [25]. In addition, six motion regressors from realignment and a CSF-based nuisance regressor were included, the latter having been shown to improve sensitivity in functional BOLD studies [36]. We deliberately did not regress out white matter or global signals, as global signal regression might introduce spurious anti-correlations and distort estimates of effective connectivity, which is particularly problematic for DCM where both the sign and magnitude of coupling parameters carry mechanistic interpretation [36].

### Participant exclusion

Since the presence of outliers and artifacts in large multi-centric brain-behavioural studies can result in biased effects in group level models, we excluded severe outliers based on the following criteria:

1. more than eight errors in indoor/outdoor judgements during fMRI;
2. an absolute response bias greater than 1.5 in the post-fMRI recognition test; and.
3. framewise displacement (FD) exceeding 0.5 mm for any frame or 0.2 mm for at least 2% of frames.

This led to the exclusion of 65 participants (17 CN, 18 SCD, 18 MCI, 12 DAT), representing 11.6% of the original sample.

### Dynamic Causal Modelling (DCM)

To investigate ATN pathology-related connectivity changes in the temporo-parietal network in the context of the visual memory-encoding paradigm, we implemented Dynamic Causal Modelling (DCM) using SPM12, following the framework described by [11]. DCM was employed to estimate effective connectivity parameters at the individual level using a Bayesian approach.

#### Region of interest specification and time-series extraction

**Fig. 2:**
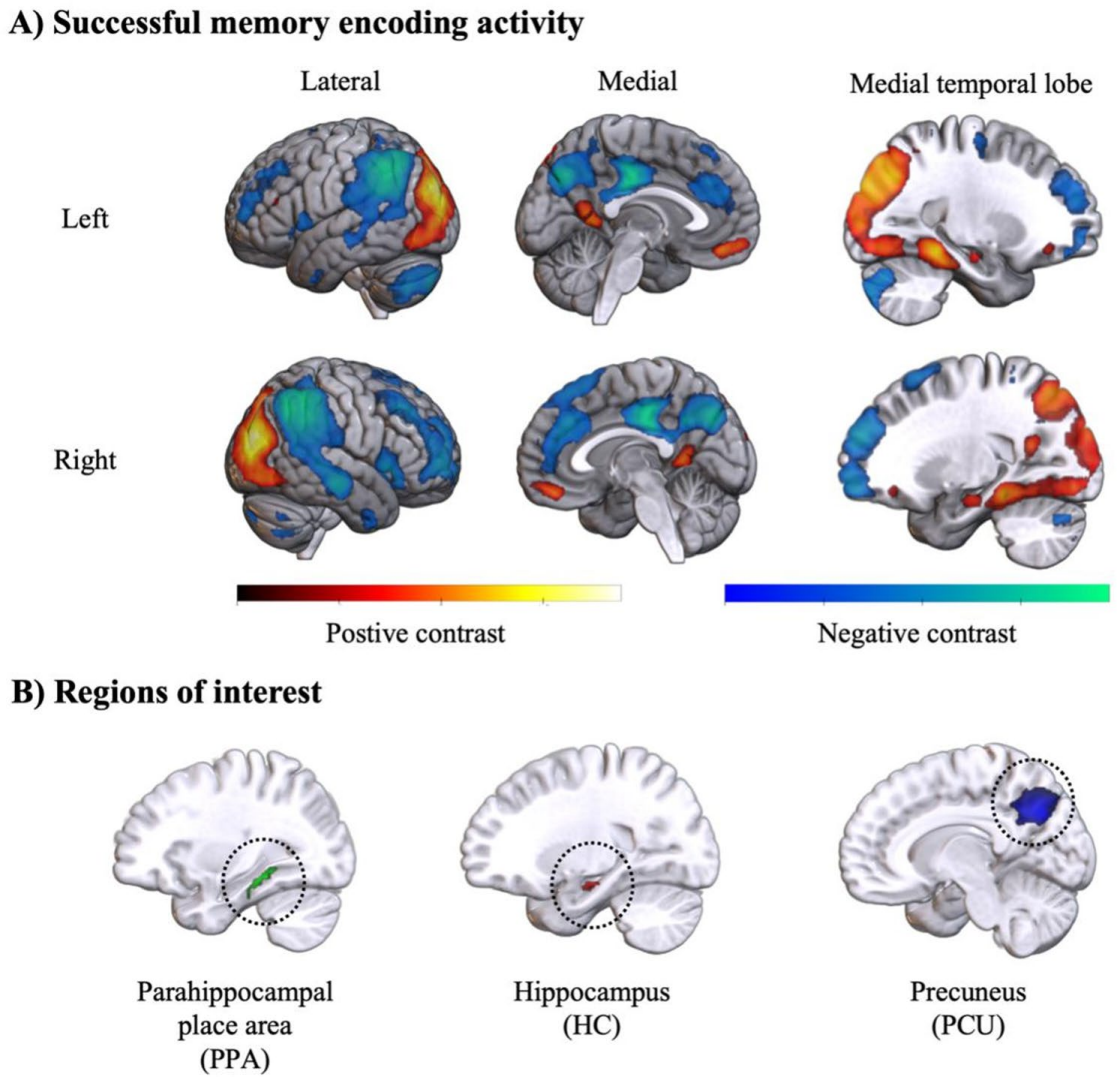
Memory contrast and regions of interest. **A** Successful memory-encoding contrast indicating fMRI BOLD signal activations (red/yellow) and deactivations (blue/green) during the encoding of a visual scene that was later successfully remembered. The contrast was derived from parametric regressor for subsequent memory score and was thresholded at *p* < 0.05, corrected for family-wise error (FWE), and controlled for Alzheimer’s disease pathology and covariates. **B** DCM Regions of interest used for time-series data extraction obtained from overlapping significant successful memory-encoding contrast (*p* < 0.05 FWE) from (**A**) and anatomically defined mask (see methods). Abbreviations: PPA (parahippocampal place area), HC (hippocampus), PCU (precuneus)

In order to study AD effects on functional dynamics during visual encoding memory-encoding paradigm we focused on brain regions of interest (ROI) including the PPA, the HC, and the PCU (Fig. 2). Despite more brain being potentially involved in the neurocognitive process [37], we deliberately limited the particular regions included in the model in order to ensure the feasibility of model estimation, as increasing the number of regions leads to quadratic growth in model parameters while sample size is fixed and limited [38]. To do so, we explored the relevant regions identified through the mutual findings between our memory contrast map from 203 CN individuals (Supplementary Table 1) and cross-modality meta-analysis of fMRI studies involving subsequent memory and forgetting [37]. These three ROIs were chosen based on earlier studies demonstrating their pronounced role in successful memory encoding [37] and their sensitivity to changes in relation to aging [39] and AD pathology [19, 22] Moreover, DCM using these ROIs has previously been successfully applied to the same visual memory-encoding paradigm in independent cohorts of healthy older adults [17]. Note that, although the brain activity during visual memory encoding is symmetrical in the broader picture [37], we chose to analyse the ROIs in the right hemisphere because of the greater activity observed in our study.

As suggested previously the boundaries of each ROI were defined as the overlapping area between anatomical and functional constraints. Anatomical constraints were based on masks from the Automated Anatomical Labelling (AAL) atlas [40]. Notably, we assume that high-quality diffeomorphic registration brings participants into sufficiently close alignment such that residual interpolation error is negligible relative to measurement and model noise. Empirically, DCM parameters show good test–retest reliability under modest spatial transformations [41]. Moreover, prior studies commonly warp into MNI space [38] and modern DCM pipelines (e.g. in Frässle et al. [42]) explicitly perform normalization before model inversion.

Functional constraints were defined using positive memory contrasts for PPA and HC, and negative memory contrasts for PCU, corresponding to our memory contrast findings and other studies [19, 26, 39, 43–45]. These constraints were derived from second-level group-level memory contrasts of the entire CN subgroup in the DELCODE cohort (*n* = 203 ; *p* < 0.05, FWE-corrected), controlling for age, sex, and education. We omitted individual-level voxel thresholding empirically due to subgroup-dependent variability of available voxels in relation to hippocampal volume and memory performance (*p* < 0.05 for both; see also [46]). Time-series data in these ROIs were extracted and adjusted to regress out known confounds and retain the effects of interest (EOI), namely the regressors representing the novel images, their parametric modulation with the subsequent memory scores [22, 26], and the pre-exposed images. Finally, the time-series data were summarised in terms of their first principal component across voxels, which formed the data entering the DCM analysis.

#### Model specification and selection

The three ROIs were assumed to be fully bidirectionally inter-connected. Each ROI was assumed to exhibit inhibitory self-connectivity reflecting local excitation-inhibition balance between (glutamatergic) pyramidal neurons and (GABAergic) interneuron [11, 47]. Presentation of novel stimuli served as the driving input to the PPA due to its selective response to novel scenes [48–50]. The model space further allowed each connection to be modulated by arcsine-transformed recognition responses (a proxy for successful encoding on a trial-by-trial basis) [25]. Because the modulatory pattern was unknown, we evaluated models using Bayesian model reduction (BMR) followed by Bayesian model averaging (BMA), as implemented in SPM12 [51]. We first specified a full model with all connections modulated by memory encoding, then compared it with reduced models generated by a greedy-search algorithm. Parameter estimates were averaged across models weighted by log-evidence differences. Modulatory units were retained if their posterior probability (Pp) ≥ 0.95 (Supplementary Table 2) [52].

### Model inversion

Model estimation was performed using SPM12 [11]. Single-subject DCMs were estimated using variational Laplace [53, 54], providing posterior connectivity estimates and free-energy approximations to the marginal likelihood. Novelty (i.e., presentation of novel, but not pre-familiarized images) served as driving input to the PPA, and encoding success (arcsine-transformed recognition responses) was included as a contextual modulator as derived from Bayesian model averaging. Neural responses to pre-familiarised images served as the implicit baseline [17]. As the sign of the estimated DCM parameters is not equated with cellular-level excitation or inhibition, it is reported as positive or negative effective connectivity.

### Evaluating effects of AD pathology on effective connectivity

We next investigated the potential effects of Aβ42/40 and p-tau-181 on connectivity using the parametric empirical Bayes (PEB) framework as implement in SPM12 [51]. To account for potentially non-linear relationships as suggested in previous studies [1, 19–21], we introduced the interaction term of amyloid status (A) and p-tau-181 level in the evaluation as such:

$$\begin{aligned}\:EC\:\sim&\:A+p-tau\:+\:A\:x\:(p-tau)\\&+age+\:sex+education\end{aligned}$$

The binary amyloid variable was introduced in the interaction term to facilitate interpretability (see a similar approach in Düzel et al. [20]). The interaction term between p-tau-181 and amyloid status captures the main effect of p-tau-181 within each amyloid stratum. Age, sex and education were included as linear covariates. The effects were evaluated using BMA with a greedy-search algorithm. We present the results with posterior probability (Pp) ≥ 0.95 [52].

Although site could be included to account for the multicentre study design, such site-effects and potential variations across MRI scanners, it was not included since it was identified to be confounded with levels of pathology (Kruskal-wallis age *p* = 0.003; education *p* = 0.003; hippocampal volume *p* = 0.003; memory performance *p* = 0.005). In addition to the primary results, we re-estimated the PEB model with imaging site included as a covariate. Hippocampal volume was not included as a covariate because it represents a downstream consequence of the same neurodegenerative processes under investigation; controlling for it would partial out variance attributable to neurodegeneration and thus obscure the very pathology–connectivity relationship the model aims to capture. Diagnosis and its interaction with biomarkers were not included in the model to avoid excessive complexity and potential collinearity.

### Identifying associations of connectivity and memory performance

To determine if the pathological connectivity changes are transferred into cognitive decline, we evaluated partial correlation of individual DCM parameters in relation to memory performance during the fMRI task itself, with age, sex, and education as covariates. Given the anticipated non-linearity of these relationships [1, 19, 21], Spearman’s rank correlation was employed. Next, correlations were computed with the PACC5 score as an independent measure of memory performance (distributions across clinical diagnoses are shown in Supplementary Fig. 1). For correlation, we used the whole subgroup with fMRI data in the DELCODE cohort (*n* = 492). The correlation was corrected for multiple comparisons using the false discovery rate (FDR) with *p* < 0.05 [55].

To verify whether these relationships also generalised to cognitive measures independent of the fMRI task, we performed out-of-sample estimations of memory performance using intrinsic and modulatory connectivity parameters derived from DCM, applying leave-one-out cross-validation within the parametric empirical Bayes (PEB) framework implemented in SPM12 [51].

## Results

### Participants and clinical characteristics

We analysed data from 492 older participants in the multicentre DELCODE cohort (see Methods for exclusion criteria), including 203 CN, 203 SCD, 65 MCI, and 21 DAT participants. The sample was 53.25% female, with a mean age of 69.76 ± 5.66 years. A subset of 205 individuals, comprising 87 CN, 89 SCD, 22 MCI, and 7 DAT participants (47.23% female; mean age = 69.03 ± 5.17 years), were categorised as A-T-N- (*n* = 150), A + T-N- (*n* = 35), or A + T+N- (*n* = 20) and were used for biomarker-connectivity analyses. 30 participants with CSF who had N+ status were excluded to isolate the A→T cascade. Demographic and clinical characteristics, including biomarker distributions, are summarised in Table 1.

**Table 1:** Characteristics of the samples in the study by analyses

Biomarker-Connectivity relationship (*n* = 205)					
	CN	SCD	MCI	DAT	all
*n*	87	89	22	7	205
ATN category (A-T-N-/A + T-N-/A + T+N-)	70/14/3	66/15/8	13/4/5	1/2/4	150/35/20
age (yr)	67.66 (4.82)	69.99 (5.40)	70.59 (4.37)	69.05 (5.45)	69.03 (5.17)
Sex (% female)	54.02	40.45	36.36	85.71	47.32
education (yr)	14.44 (2.69)	15.28 (2.94)	13.68 (2.34)	13.71 (1.60)	14.70 (2.78)
Memory performance	0.76 (0.08)	0.77 (0.09)	0.69 (0.09)	0.61 (0.07)	0.75 (0.09)
PACC5	0.13 (0.54)	-0.14 (0.68)	-1.13 (0.80)	-3.07 (1.36)	-0.20 (0.88)
Aβ42/40 ratio	0.10 (0.02)	0.10 (0.03)	0.09 (0.03)	0.05 (0.02)	0.09 (0.03)
p-tau (pg/ml)	46.78 (14.49)	52.87 (23.17)	58.15 (34.71)	106.24 (66.02)	52.68 (26.25)
Hippocampal volume	3698.09 (392.83)	3706.99 (423.12)	3447.05 (376.65)	3307.53 (226.27)	3661.68 (411.25)

Connectivity-Memory performance correlation (*n* = 492)					
	**CN**	**SCD**	**MCI**	**DAT**	**all**
*n*	203	203	65	21	492
age (yr)	68.17 (5.14)	70.06 (5.89)	72.63 (4.76)	73.36 (5.38)	69.76 (5.66)
Sex (% female)	61.58	43.84	52.31	66.67	53.25
education (yr)	14.55 (2.72)	15.26 (2.90)	13.43 (2.81)	13.71 (2.80)	14.66 (2.87)
Memory performance	0.76 (0.08)	0.76 (0.09)	0.69 (0.10)	0.60 (0.08)	0.75 (0.09)
PACC5	0.19 (0.59)	-0.05 (0.71)	-1.22 (0.79)	-2.97 (1.18)	-0.20 (0.97)

The table provides an overview of the characteristics of the subsamples used in the study. The upper section details the subsample examined using the parametric empirical Bayes (PEB) to evaluate the relationship between ATN biomarkers and effective connectivity. The lower section summarises the subsample analysed with Spearman’s rank correlation to assess the relationship between effective connectivity and memory performance. Summary statistics are reported as means, with standard deviations in parentheses. Abbreviations: *CN *cognitively normal, *SCD *subjective cognitive decline, *MCI *mild cognitive impairment, and *DAT *dementia of Alzheimer’s type

### Dynamic causal model of memory encoding

**Fig. 3:**
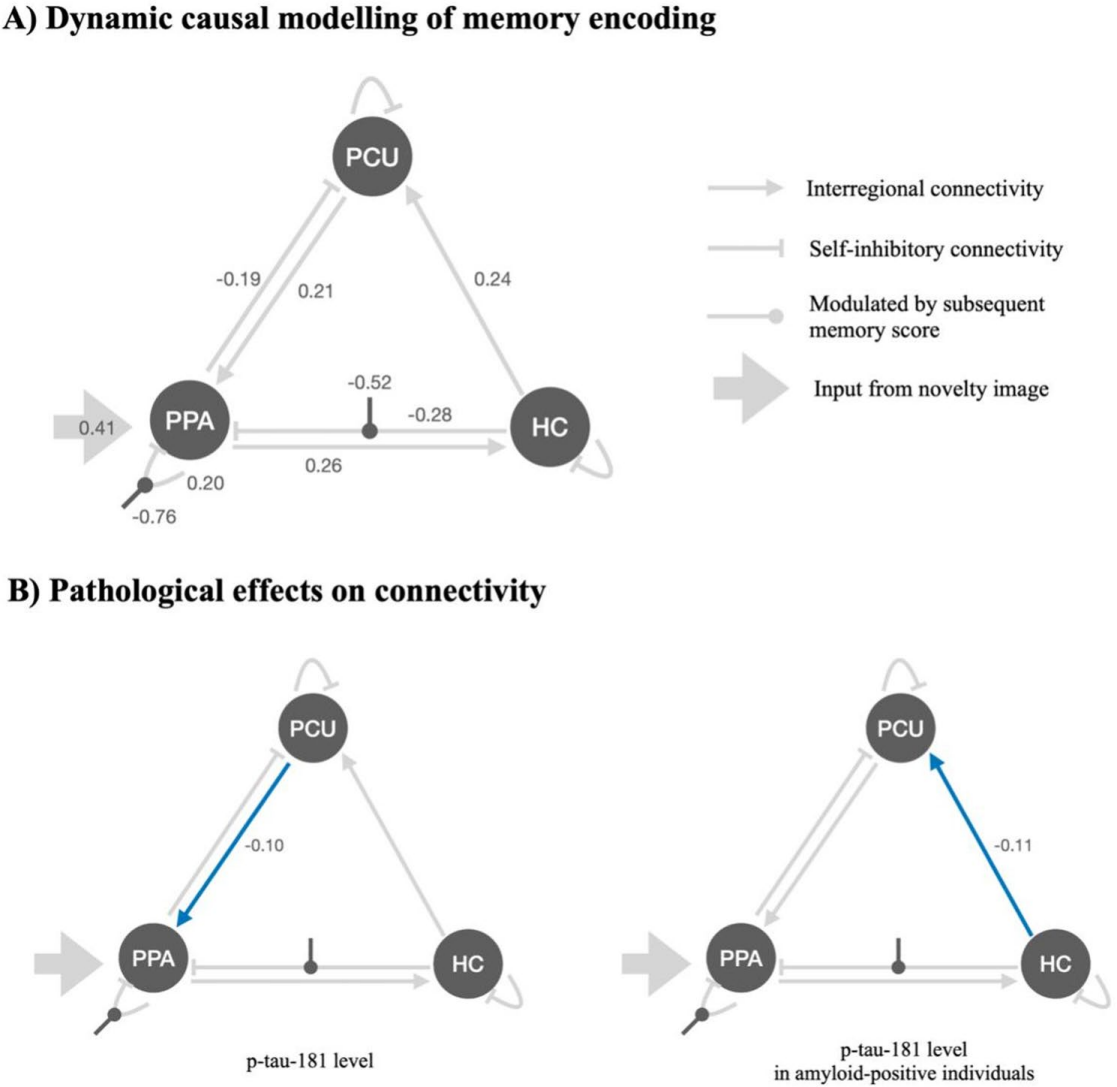
Dynamic causal model of memory encoding and the effect of Alzheimer’s pathology on the model. **A** Dynamic causal model of memory-encoding task fMRI obtained through model Bayesian model average of memory-related modulation in 203 cognitively normal older participants (ages 60–80 years), as seen in the round-headed arrows. Effective connectivity coefficients are also displayed on the arrows that represent model parameters. **B** Effects of p-tau-181 level and p-tau-181 level in amyloid-positive individual are highlighted in blue. Abbreviations: PPA (parahippocampal place area), HC (hippocampus), PCU (precuneus)

To explore the influence of ATN pathology on memory-related effective connectivity in ageing and Alzheimer’s disease, we applied DCM to fMRI data acquired during a visual memory-encoding task, focusing on a network comprising the PPA, HC, and PCU (Fig. 2B; see also Methods). Individual-level DCMs were estimated for all participants, and Fig. 3A illustrates the mean group-level connectivity profile obtained from the parametric empirical Bayes (PEB) approach together with pathological effect (Supplementary Table 3). The connectivity with Pp > 0.95 is reported.

For task-related intrinsic connectivity, connections from PPA → PCU and from HC → PPA were negative, whereas all other intrinsic connections were positive. Under the model’s assumptions, this indicates that during the task, PPA activity suppresses PCU activity, and HC activity effectively inhibits the PPA. Conversely, activity in the source region facilitates activity in the target region for all other connections.

DCM further allows the functional dynamics of memory encoding to be examined in terms of stimulus-driven inputs and memory-related modulations of connectivity. The driving input had a positive effect on PPA activity, indicating that PPA responses increased with novelty. Moreover, successful encoding was associated negatively with both the negative effective self-coupling of the PPA and the negative effective connectivity from HC → PPA. Together, these findings suggest that successful encoding depends on (1) stronger negative feedback from HC to PPA, and (2) optimal gain control of PPA responsiveness to stimuli (see [11] for interpretation).

### Relationship between AD pathology and connectivity

To assess how AD pathology contributes to connectivity disruption, we examined the effects of amyloid status and tau (p-tau-181) on effective connectivity using PEB that accounts for potential interactions (full results are shown in Supplementary Table 3). We observed a significant association between p-tau-181 levels and the distal intrinsic positive effective connectivity between the temporal and the parietal lobes (Fig. 3B). Specifically, higher p-tau-181 concentrations were linked to reduced connectivity strength from PCU → PPA (coef. = -0.10, Pp = 1.00). Similarly, the attenuation in the connectivity from HC → PCU was associated with higher p-tau-181 level. Notably, the latter effect was identified exclusively in amyloid-positive (A+) individuals, as indicated by the significant interaction between amyloid status and p-tau-181 levels (coef. = -0.11, Pp = 1.00), rather than p-tau-181 alone (coef. = 0.05, Pp = 0.60).

We additionally re-estimated the PEB with imaging site included as a covariate (Supplementary Table 4). The two principal pathology effects were preserved (PCU → PPA p-tau-181 effect: coef. = −0.12, Pp = 1.00; HC → PCU amyloid status and p-tau-181 interaction: coef. = −0.11, Pp = 1.00), confirming that the findings are not driven by inter-site or inter-scanner heterogeneity. The site-adjusted model additionally yielded a supra-threshold amyloid status and p-tau-181 interaction on PCU → PPA (coef. = 0.12, Pp = 1.00) that was sub-threshold in the primary model (0.09, Pp = 0.80). As imaging site is collinear with pathology, the basis for excluding it from the pre-specified primary model, coefficients that differ between specifications cannot be unambiguously attributed and are therefore not further interpreted. Only the two effects stable across both specifications are interpreted.

### Association of connectivity and memory impairment

**Fig. 4:**
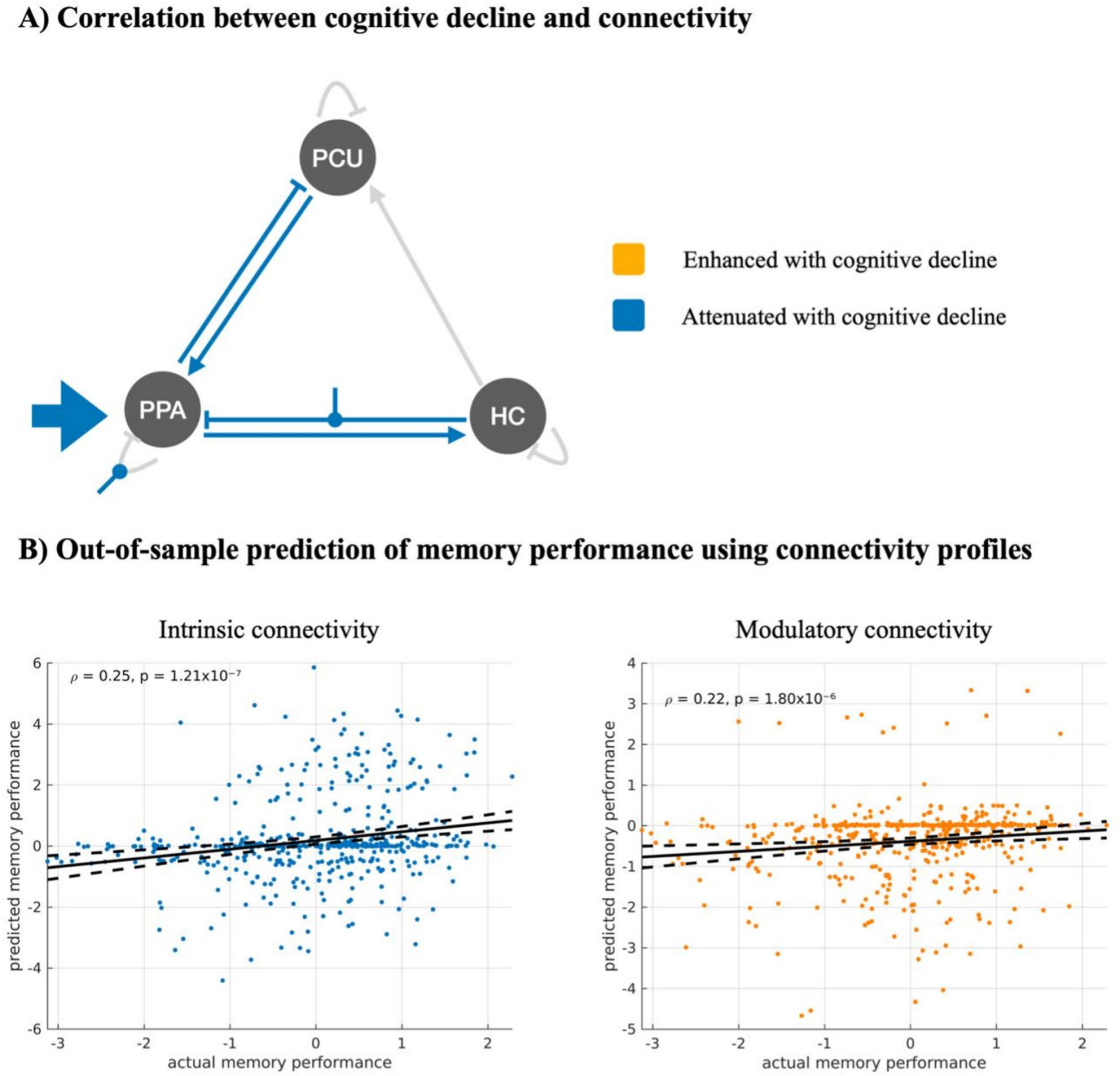
Association of the effective connectivity with memory performance. **A** Uniform reduction in connectivity is correlated to lower memory performance (positive correlation in positive effective connectivity and negative correlation in negative effective connectivity). Significant relationships were derived from partial Spearman’s rank correlations and defined by *p* < 0.05 (FDR corrected). **B** The association of predicted memory performance using connectivity profiles in out-of-sample cross-validation. Left: Using the entire intrinsic connectivity profile (DCM’s A parameters), a significant positive correlation (ρ = 0.25, *p* = 1.21 × 10^− 7^) indicates an association between memory performance and intrinsic connectivity dynamics. Right: Using the modulatory connectivity profile (DCM’s B parameters), modulatory connectivity significantly predicts memory performance (ρ = 0.22, *p* = 1.80 × 10^− 6^), further indicating a meaningful association. The results were derived from Spearman’s rank correlations. Abbreviations: PPA (parahippocampal place area), HC (hippocampus), PCU (precuneus)

We next examined associations between effective connectivity parameters and individual memory performance, quantified by the area under the curve (AUC, A′) of the delayed recognition task (performed 90 min after the fMRI session; see Methods). Partial Spearman’s rank correlations identified seven connectivity parameters that showed reduced connection strength with poorer memory performance (Fig. 4A and Supplementary Table 5).

We found that the disrupted connectivity attributed to AD pathology was partially related to cognitive decline. Specifically, the positive effective connection from PCU → PPA was found to be positively correlated with memory performance (ρ = 0.14, *p* = 5.02 × 10⁻³) implying that its attenuation is associated with reduced memory performance. On the other hand, the connectivity from HC → PCU was not significantly correlated with memory performance (ρ = 0.10, *p* = 0.06). However, the correlation was statistically significant when using uncorrected p-value (*p* = 0.03).

Between the PPA and the PCU, higher memory performance was associated with stronger bidirectional coupling: the negative effective connection from PPA → PCU was found to be more negative (ρ = -0.21, *p* = 1.56 × 10⁻⁵) while the positive effective connection from PCU → PPA was more positive (ρ = 0.14, *p* = 5.02 × 10⁻³). Between the PPA and the HC, the positive effective PPA → HC connection correlated positively with memory performance (ρ = 0.18, *p* = 4.09 × 10⁻⁴), whereas the negative effective HC → PPA connection correlated negatively (ρ = − 0.16, *p* = 1.22 × 10⁻³). In addition, the memory-related modulation of the HC → PPA pathway was negatively associated with performance (ρ = − 0.22, 7.35 × 10⁻⁶). Within the PPA itself, memory performance was positively associated with the novelty-driven input (ρ = 0.25, *p* = 3.00 × 10⁻⁷) but negatively with the memory-related modulation of negative effective self-coupling (ρ = − 0.22, *p* = 7.35 × 10⁻⁶). These effect sizes (ρ ≈ 0.14–0.25) are in the small-to-medium range by conventional standards; however, they are consistent with what is typically observed in large neuroimaging cohorts where individual variability is high and the relationships between brain measures and behaviour are inherently noisy [19, 22].

Importantly, the effects survived FDR correction and were independently replicated using the PACC5 composite score in PPA input (ρ = 0.16, *p* = 1.26 × 10⁻³), PPA → HC (ρ = 0.19, *p* = 2.61 × 10⁻4), and PPA → PCU (ρ = -0.16, *p* = 1.26 × 10⁻³), supporting the importance of forward connectivity in cross-task cognitive performance.

Finally, out-of-sample cross-validation confirmed the predictive validity of these connectivity profiles (Fig. 4B). Predicted values for both memory performance correlated significantly with observed scores based on intrinsic (ρ = 0.25, *p* = 1.21 × 10 − 7) and modulatory connectivity (ρ = 0.22, *p* = 1.80 × 10 − 6). Together, these results demonstrate that effective connectivity patterns—particularly those centred on the PPA—robustly capture inter-individual differences in memory and general cognitive performance.

## Discussion

This study employed a generative DCM approach to investigate the network dynamics underlying impaired memory encoding across the AD spectrum, aiming to elucidate the connectivity disruptions that are associated with the complex amyloid and tau pathology. Our findings revealed an association between connectivity strength in the distal connectivity between the temporal and parietal lobes and AD molecular pathology. Specifically, a tau-driven decrease in connectivity from PCU → PPA, together with a decrease in connectivity from HC → PCU—the latter amplified by amyloid—was identified as a key network disruption. Furthermore, we identified connectivity associated with memory impairment, which was restricted to PCU → PPA. This is consistent with a pathway in which molecular pathology relates to memory impairment, although the cross-sectional design cannot establish mediation.

### Regionally specific influence of amyloid and tau pathology

Amyloid and tau are well-established biomarkers of AD and have both been shown to contribute to synaptic dysfunction and neurodegeneration [2, 56, 57]. Importantly, they likely show a non-linear interaction in their contribution to AD progression: While amyloid accumulation is associated with few symptoms on its own, it plays a critical role in promoting pathological tau accumulation and influences the spatial distribution of tau by facilitating its migration from the medial temporal lobe (MTL) to the neocortex [8, 58–61]. On the other hand, tau appears to be more directly involved in the disruption of brain activity and cognition [20, 62–64].

Previous PET studies have demonstrated distinct anatomical patterns of amyloid and tau deposition [65–69]. Specifically, Aβ deposition in AD typically begins in the posterior cingulate cortex and connected parietal cortices [66, 70], while tau pathology originates in the MTL, including the entorhinal and parahippocampal cortex [71]. This dissociation is, to some extent, mirrored by the observed differential influence of tau and amyloid pathology on the temporo-parietal network. Specifically, decreased connectivity from HC → PCU was linked to elevated p-tau-181 levels and was amplified by the presence of amyloid accumulation (A+ status), assimilating the aforementioned tau spreading. Although medial temporal tau burden is the most reliable predictor of episodic memory performance independent of amyloid statu [62], both Düzel et al. [20] and our study demonstrate that amyloid modulates these functional associations. On the other hand, attenuation of the backward connectivity from PCU → PPA was linked to elevated p-tau-181 levels without the influence of amyloid status. The finding encompasses the local effect of tau accumulation which causes aberrant activity in memory encoding, corresponding to previous findings on memory-related activation [65, 72].

It should be noted, however, that the cross-sectional design of the present study does not permit causal inference regarding the temporal ordering of these effects. It remains possible that pre-existing connectivity deficits facilitate further tau accumulation and spread, rather than tau driving connectivity loss unidirectionally. Longitudinal data e.g. using time-lagged modelling might be helpful to disambiguate these competing accounts. In addition, due to the focus on the effects of amyloid and tau and the restriction to neurodegenerative change, the study was inherently weighted towards healthy and preclinical AD. While this is appropriate for a study of network disruption across the AD spectrum, the results should not be extrapolated to moderate or severe dementia stages without further validation.

### Disrupted input-gated connectivity is linked to memory performance

Considering the roles of the connectivity, these connections between PPA and the other two regions serve as gated-feedback loops comprising forward signal-enhancing activity and backward signal-suppressing activity. We found that (Fig. 4), individuals with poorer memory showed weaker top-down suppression of PCU by PPA and a reciprocal facilitation of PPA by PCU (interpreted as such because of the deactivation pattern of PCU). Similarly, individuals with lower memory performance exhibited reduced PPA-driven facilitation of hippocampal activity and diminished HC-mediated negative effective self-coupling of PPA. The findings entail an attenuation of the feedback-loop structure, which can serve as an explanation for network disconnection in AD [65–74], where we have identified an effect of p-tau-181 on the connectivity PCU → PPA. Noticeably, the correlation to cognitive decline was replicated using the PACC5 composite score in the forward connectivity PPA → HC and PPA → PCU. The common pattern suggests the importance of forward connectivity in cross-task cognitive performance, while it also emphasises the specific function of the feedback loop in memory-encoding.

### Limitations

This study has several limitations. First, the proportion of participants in later disease stages of the AD spectrum was relatively low (*n* = 21 DAT in the full sample; *n* = 7 from ATN classification of interest), which may have limited the ability to detect biomarker effects in the manifest clinical stages of the disease. The resulting sample imbalance—with CN and SCD comprising ~ 83% of the cohort—means that the observed connectivity–memory correlations are predominantly driven by preclinical and early symptomatic stages, as seen in the marginally significant correlation between HC → PCU, which is expected in A+ stages. This limitation was further compounded by the challenges faced by participants with dementia in complying with task-based fMRI protocols, resulting in their exclusion from the study. Second, we were unable to extract time-series data using a threshold for significant voxel activity at the single-subject level. This limitation arose because the absence of significant voxels was associated with reduced hippocampal volume and poorer memory performance. As recently shown, individuals in the later stages of the AD spectrum exhibit diminished fMRI subsequent memory effects [47], which would have resulted in a systematic exclusion of individuals at later stages. Aware of the trade-off between data quality and inclusivity, we prioritised a comprehensive representation of the full AD spectrum, ensuring that the characteristics of the later stages were captured. Third, the CSF biomarkers used in this study lacked spatial specificity, which is informative for understanding complex interactions involving regionally specific aggregation and protein migration. Moreover, we did not examine alternative tau species such as p-tau-217 or p-tau-231, which have recently demonstrated superior diagnostic sensitivity in some contexts, nor blood-based biomarkers (e.g., plasma p-tau-217, GFAP) that would enhance translational relevance. Incorporating these markers in future analyses, as they become available within the DELCODE cohort, will be an important extension of this work. Fourth, our DCM comprised three ROIs, deliberately excluding regions such as the entorhinal cortex or prefrontal cortex. While this parsimonious approach ensured stable model estimation and was grounded in prior work [17], it necessarily limits the completeness of the network characterisation. We note that exploratory analyses with larger model spaces did not yield meaningful additional effects, possibly due to the combinatorial growth of model parameters relative to the data [38]. Future work using regression DCM45 or hierarchical models may permit the inclusion of additional nodes. Finally, there are several concerns regarding DCM, including a limited external validation, potential model simplification, and strong assumptions [75, 76]. To address these issues, we implemented several procedures, such as adopting theory-driven models and enhancing transparency through cross-validation methods. Importantly, the cross-sectional nature of this study precludes causal conclusions about whether AD pathology drives connectivity disruption or vice versa; longitudinal follow-ups and interventions might be helpful to establish evidence about hypothesises of directionality.

## Conclusions

In conclusion, this study provides novel insights into the disrupted connectivity with which Alzheimer’s pathology is associated, and which relates to memory performance, emphasising the importance of the temporo-parietal network during memory encoding in AD. Specifically, the findings highlight that decreased connectivity from HC → PCU is associated with elevated p-tau-181 levels and amplified by amyloid accumulation. While backward connectivity from PCU → PPA was only associated with p-tau-181, independent of amyloid status, emphasising the asymmetric pathology between temporal and parietal regions. By identifying specific connectivity pathways linked to Alzheimer’s biomarkers, this work opens new avenues for targeted interventions aimed at mitigating memory deficits through the preservation or restoration of synaptic function.

## Supplementary Information

Supplementary Material 1.

## Data Availability

Data, study protocol, and biomaterials can be shared with partners based on individual data and biomaterial transfer agreements. Access to the relevant study data can be obtained by submitting an application to the Clinical Research Platform of the DZNE (https:/www.dzne.de/en/research/research-areas/clinical-research/for-researchers).

## Abbreviations

Aβ: Beta-amyloid
AD: Alzheimer’s disease
BMA: Bayesian model averaging
BMR: Bayesian model reduction
CN: Cognitively normal
DAT: Dementia of Alzheimer’s type
DCM: Dynamic causal modelling
DELCODE: DZNE - longitudinal cognitive impairment and dementia study
DZNE: German centre for neurodegenerative diseases
HC: Hippocampus
MCI: Mild cognitive impairment
PEB: Parametric empirical Bayes
PPA: Parahippocampal place area
PCU: Precuneus
p-tau: Phosphorylated tau
SCD: Subjective cognitive decline
