# Supplementary material for: Disruption of temporo-parietal network in Alzheimer’s disease and its association with memory impairment
Source: Alzheimers Res Ther. 2026 Jul 15;18:166. doi: 10.1186/s13195-026-02138-w (PMC13374064; doi:10.1186/s13195-026-02138-w)
Supplement: Supplementary file 1 — Supplementary Material 1. [file 13195_2026_2138_MOESM1_ESM.docx]

# **Supplementary material**

## **Supplementary Table 1 Regions activating during the memory-encoding task.**

| **Region** | **Hemisphere** | **Coordinate** | | | **corresponding AAL parcellation** |
| --- | --- | --- | --- | --- | --- |
|  |  | **X** | **Y** | **Z** |  |
| **Positive memory contrast** | | | | | |
| Hippocampus | Left | -21 | -14 | -17.5 | Hippocampus_L |
| Fusiform cortex/parahippocampus | Left | -24.5 | -42 | -14 | Fusiform_L |
| Occipital lobe | Left | -31.5 | -80.5 | 28 | Occipital_Mid_L |
| Precuneus | Left | -7 | -56 | 14 | Precuneus_L |
| Orbital frontal cortex | Left | -3.5 | 42 | -17.5 | Rectus_L |
| Inferior frontal gyrus | Left | -31.5 | 31.5 | -14 | Frontal_Inf_Orb_L |
| Inferior frontal gyrus | Left | -42 | 7 | 28 | Frontal_Inf_Oper_L |
| Hippocampus | Right | 21 | -14 | -17.5 | ParaHippocampal_R |
| Fusiform cortex/parahippocampus | Right | 31.5 | -38.5 | -14 | Fusiform_R |
| Occipital lobe | Right | 35 | -80.5 | 21 | Occipital_Mid_R |
| Precuneus | Right | 10.5 | -52.5 | 10.5 | Precuneus_R |
| Orbital frontal cortex | Right | 0 | 49 | -17.5 | Rectus_L |
| Inferior frontal gyrus | Right | 31.5 | 35 | -10.5 | Frontal_Inf_Orb_R |
| Inferior frontal gyrus | Right | 38.5 | 7 | 28 | Frontal_Inf_Oper_R |
| **Negative memory contrast** | | | | | |
| Inferior parietal lobule | Left | -63 | -49 | 28 | SupraMarginal_L |
| Middle temporal gyrus | Left | -59.5 | -24.5 | -10.5 | Temporal_Mid_L |
| Precuneus | Left | -7 | -70 | 35 | Precuneus_L |
| Mid cingulate cortex | Left | 0 | -24.5 | 35 | Cingulum_Mid_L |
| Anterior cingulate cortex | Left | -3.5 | 31.5 | 17.5 | Cingulum_Ant_L |
| Middle frontal gyrus | Left | -38.5 | 42 | 21 | Frontal_Mid_L |
| Rolandic opreculum | Left | -52.5 | 10.5 | 3.5 | Rolandic_Oper_L |
| Inferior parietal lobule | Right | 56 | -52.5 | 42 | Parietal_Inf_R |
| Middle temporal gyrus | Right | 63 | -21 | -14 | Temporal_Mid_R |
| Precuneus | Right | 10.5 | -66.5 | 31.5 | Precuneus_R |
| Mid cingulate cortex | Right | 0 | -24.5 | 35 | Cingulum_Mid_L |
| Anterior cingulate cortex | Right | 7 | 38.5 | 17.5 | Cingulum_Ant_R |
| Middle frontal gyrus | Right | 28 | 52.5 | 21 | Frontal_Mid_R |
| Inferior frontal gyrus | Right | 52.5 | 14 | 3.4 | Frontal_Inf_Oper_R |
| Supplementary motor area | Right | 14 | 14 | 63 | Supp_Motor_Area_R |

The table provides coordinates of regional activity from memory contrast from 203 CN individuals (p<0.05 FWE, cluster threshold > 10) with the corresponding ROIs in AAL parcellation obtained from the peak coordinate.

## **Supplementary Table 2 Expected posterior and posterior probability of modulatory connectivity obtained from Bayesian model average.**

| **Connectivity** | **expected posterior** | **posterior probability**  **(\|Ep\|>0 )** |
| --- | --- | --- |
| PPA to PPA | -0.96 | 1.00 |
| PPA to HC | 0.09 | 0.50 |
| PPA to PCU | 0.00 | 0.00 |
| HC to PPA | -0.28 | 0.96 |
| HC to HC | 0.00 | 0.00 |
| HC to PCU | 0.00 | 0.00 |
| PCU to PPA | 0.00 | 0.00 |
| PCU to HC | 0.00 | 0.00 |
| PCU to PCU | 0.00 | 0.00 |

## The table presents the expected posterior and posterior probability of the modulatory connectivity of memory encoding derived from Bayesian model average of the healthy individuals. Abbreviations: PPA (parahippocampal place area), HC (hippocampus), and PCU (precuneus), CN (cognitively normal), SCD (subjective cognitive decline), MCI (mild cognitive impairment), and DAT (dementia of Alzheimer's type).

## **Supplementary Table 3 Mean connectivity and the effects of amyloid-β42/40 (Aβ42/40), phospho-tau-181 (p-tau-181) on connectivity.**

| **Connectivity** | **mean** | **Amyloid status** | **p-tau-181 level** | **A status x p-tau-181 interaction** |
| --- | --- | --- | --- | --- |
| **Intrinsic** | | | | |
| PPA to PPA | 0.20 (1.00)* | 0.00 (0.00) | 0.00 (0.00) | 0.00 (0.00) |
| PPA to HC | 0.26 (1.00)* | 0.00 (0.00) | -0.00 (0.00) | 0.00 (0.00) |
| PPA to PCU | -0.19 (1.00)* | 0.00 (0.00) | 0.00 (0.00) | 0.03 (0.51) |
| HC to PPA | -0.28 (1.00)* | 0.00 (0.00) | 0.00 (0.00) | 0.00 (0.00) |
| HC to HC | 0.03 (0.54) | -0.00 (0.00) | -0.00 (0.00) | -0.00 (0.00) |
| HC to PCU | 0.24 (1.00)* | -0.00 (0.00) | 0.05 (0.60) | -0.11 (1.00)* |
| PCU to PPA | 0.21 (1.00)* | 0.00 (0.00) | -0.10 (1.00)* | 0.09 (0.80) |
| PCU to HC | 0.04 (0.68) | 0.00 (0.00) | 0.00 (0.00) | 0.00 (0.00) |
| PCU to PCU | -0.04 (0.55) | -0.00 (0.00) | 0.00 (0.00) | -0.00 (0.00) |
| **Modulation** | | | | |
| PPA to PPA | -0.76 (1.00)* | -0.02 (0.00) | -0.00 (0.00) | -0.01 (0.12) |
| HC to PPA | -0.52 (1.00)* | -0.04 (0.21) | -0.01 (0.00) | -0.02 (0.17) |
| **Input** | | | | |
| PPA | 0.41 (1.00)* | 0.01 (0.07) | -0.01 (0.07) | -0.02 (0.08) |

The table presents the effects of Alzheimer's pathology (amyloid-β42/40 and phospho-tau181) on effective connectivity. The results are displayed in coefficient and posterior probability. Statistical significance is denoted by * for *Pp>0.95*. Abbreviations: PPA (parahippocampal place area), HC (hippocampus), PCU (precuneus), Aβ42/40 (amyloid-β42/40), p-tau-181 (phospho-tau181).

**Supplementary Table 4 Mean connectivity and the effects of amyloid-β42/40 (Aβ42/40), phospho-tau-181 (p-tau-181) on connectivity, with site as covariates.**

| **Connectivity** | **mean** | **Amyloid status** | **p-tau-181 level** | **A status x p-tau-181 interaction** |
| --- | --- | --- | --- | --- |
| **Intrinsic** | | | | |
| PPA to PPA | 0.20 (1.00) * | 0.00 (0.00) | 0.00 (0.00) | 0.00 (0.00) |
| PPA to HC | 0.26 (1.00) * | 0.00 (0.00) | -0.00 (0.00) | 0.00 (0.00) |
| PPA to PCU | -0.19 (1.00) * | 0.00 (0.00) | 0.00 (0.00) | 0.03 (0.52) |
| HC to PPA | -0.28 (1.00) * | 0.00 (0.00) | 0.00 (0.00) | 0.03 (0.50) |
| HC to HC | 0.03 (0.52) | -0.00 (0.00) | 0.00 (0.00) | 0.00 (0.00) |
| HC to PCU | 0.24 (1.00) * | 0.00 (0.00) | 0.05 (0.60) | -0.11 (1.00) * |
| PCU to PPA | 0.21 (1.00) * | 0.00 (0.00) | -0.12 (1.00) * | 0.12 (1.00) * |
| PCU to HC | 0.06 (1.00) * | 0.00 (0.00) | 0.00 (0.00) | 0.00 (0.00) |
| PCU to PCU | -0.04 (0.56) | -0.00 (0.00) | 0.00 (0.00) | -0.00 (0.00) |
| **Modulation** | | | | |
| PPA to PPA | -0.75 (1.00) * | -0.03 (0.00) | -0.00 (0.00) | -0.01 (0.00) |
| HC to PPA | -0.52 (1.00) * | -0.04 (0.00) | -0.00 (0.00) | -0.02 (0.00) |
| **Input** | | | | |
| PPA | 0.41 (1.00) * | -0.00 (0.00) | -0.02 (0.07) | -0.01 (0.08) |

The table presents the effects of Alzheimer's pathology (amyloid-β42/40 and phospho-tau181) on effective connectivity. The results are displayed in coefficient and posterior probability. Statistical significance is denoted by * for Pp>0.95. Abbreviations: PPA (parahippocampal place area), HC (hippocampus), PCU (precuneus), Aβ42/40 (amyloid-β42/40), p-tau-181 (phospho-tau181).

## **Supplementary Table 5 Spearman's rank correlation of effective connectivity and memory performance.**

| **Connectivity** | **Memory performance** | **PACC5** |
| --- | --- | --- |
| **Intrinsic** | | |
| PPA to PPA | 0.06 (0.31) | 0.05 (0.31) |
| PPA to HC | 0.18 (4.09x10⁻⁴)* | 0.19 (2.61x10⁻⁴)* |
| PPA to PCU | -0.21 (1.56x10⁻⁵)* | -0.16 (1.26x10⁻³)* |
| HC to PPA | -0.16 (1.22x10⁻³)* | -0.10 (0.06) |
| HC to HC | -0.05 (0.36) | -0.08 (0.11) |
| HC to PCU | 0.10 (0.06) | 0.01 (0.87) |
| PCU to PPA | 0.14 (5.02x10⁻³)* | 0.09 (0.11) |
| PCU to HC | -0.01 (0.80) | -0.02 (0.67) |
| PCU to PCU | 0.08 (0.14) | -0.02 (0.67) |
| **Modulation** | | |
| PPA to PPA | -0.22 (7.35x10⁻⁶)* | -0.10 (0.07) |
| HC to PPA | -0.22 (7.35x10⁻⁶)* | -0.04 (0.51) |
| **Input** | | |
| PPA | 0.25 (3.00x10⁻⁷)* | 0.16 (1.26x10⁻³)* |

The table presents the Spearman's correlation coefficients (*ρ*) and p-values for each connection's relationship with memory performance and PACC5 test, with age, sex, and education as covariates. To indicate the amplitude of the effect, the significance column uses the following symbols: '*' for p < 0.05*.* P-values were adjusted for false discovery rate (FDR) using the Benjamini-Hochberg procedure. Abbreviations: PPA (parahippocampal place area), HC (hippocampus), and PCU (precuneus).

## **Supplementary Figure 1 Distribution of cognitive measurements**

**
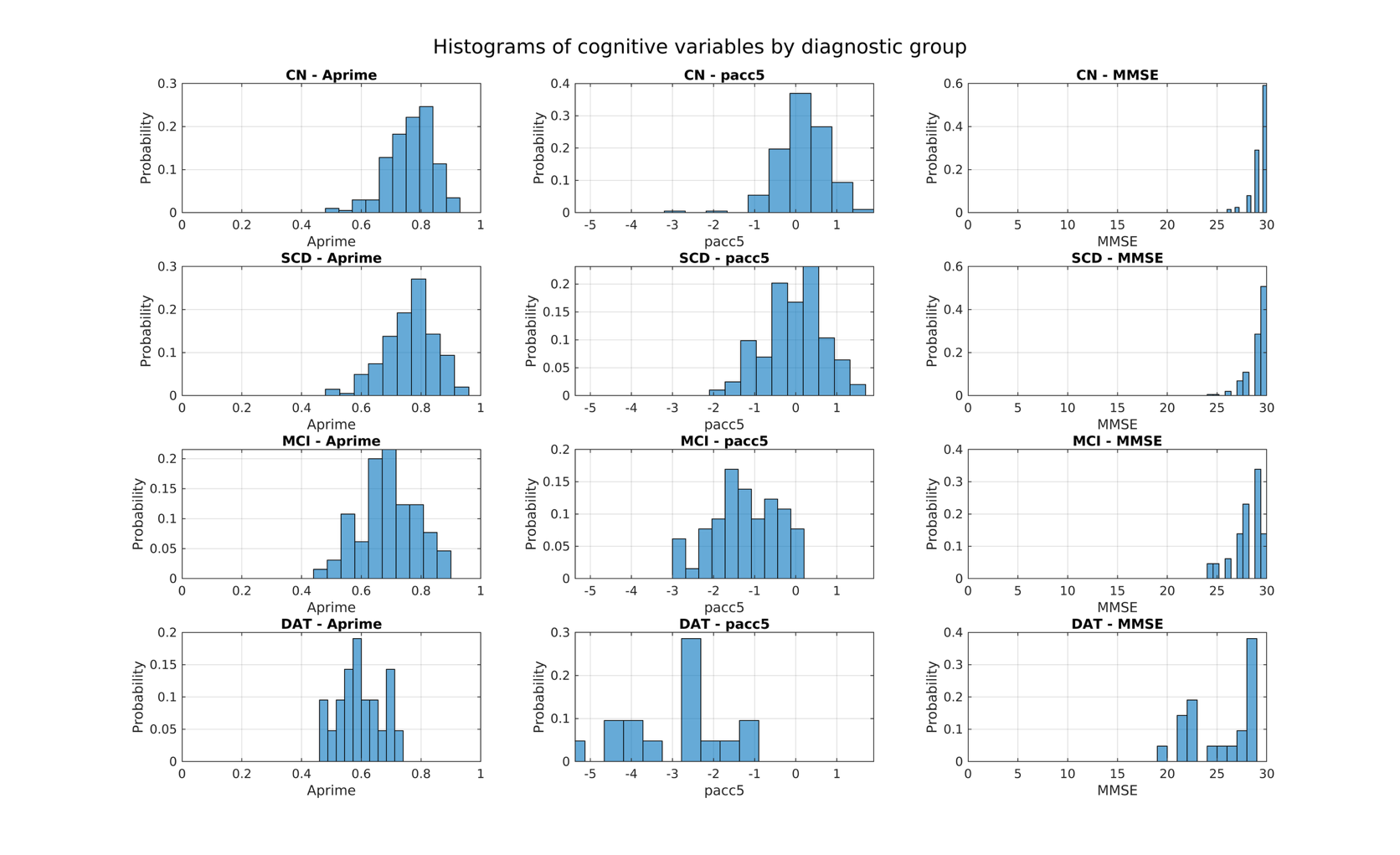
**

The histograms show the distribution of memory performance(A'), PACC5, and MMSE by diagnoses. Unlike MMSE, A′ and PACC5 show no ceiling effect across diagnostic groups.
